# Procoagulant and immunogenic properties of melanoma exosomes, microvesicles and apoptotic vesicles

**DOI:** 10.18632/oncotarget.10783

**Published:** 2016-07-22

**Authors:** Morad-Rémy Muhsin-Sharafaldine, Sarah C. Saunderson, Amy C. Dunn, James M. Faed, Torsten Kleffmann, Alexander D. McLellan

**Affiliations:** ^1^ Department of Microbiology and Immunology, University of Otago, Dunedin, Otago, New Zealand; ^2^ Department of Pathology, University of Otago, Dunedin, Otago, New Zealand; ^3^ Centre for Protein Research, University of Otago, Dunedin, Otago, New Zealand; ^4^ Department of Microbiology and Immunology, Otago School of Medical Sciences, University of Otago, Dunedin, Otago, New Zealand

**Keywords:** tumor vesicles, apoptotic vesicles, coagulation, tissue factor, cancer immunity

## Abstract

Extracellular vesicles (EV) are lipid particles released from eukaryotic cells into the extracellular fluid. Depending on the cell type or mechanism of release, vesicles vary in form and function and exert distinct functions in coagulation and immunity. Tumor cells may constitutively shed vesicles known as exosomes or microvesicles (MV). Alternatively, apoptosis induces the release of apoptotic blebs or vesicles (ApoV) from the plasma membrane. EV have been implicated in thrombotic events (the second highest cause of death in cancer patients) and tumor vesicles contribute to the anti-cancer immune response. In this study, we utilized the well characterized B16 melanoma model to determine the molecular composition and procoagulant and immunogenic potential of exosomes, MV and ApoV. Distinct patterns of surface and cytoplasmic molecules (tetraspanins, integrins, heat shock proteins and histones) were expressed between the vesicle types. Moreover, *in vitro* coagulation assays revealed that membrane-derived vesicles, namely MV and ApoV, were more procoagulant than exosomes–with tissue factor and phosphatidylserine critical for procoagulant activity. Mice immunized with antigen-pulsed ApoV and challenged with B16 tumors were protected out to 60 days, while lower protection rates were afforded by MV and exosomes. Together the results demonstrate distinct phenotypic and functional differences between vesicle types, with important procoagulant and immunogenic functions emerging for membrane-derived MV and ApoV versus endosome-derived exosomes. This study highlights the potential of EV to contribute to the prothrombotic state, as well as to anti-cancer immunity.

## INTRODUCTION

Extracellular vesicles (EV) are lipid bilayer-enclosed particles released by most mammalian cell types [1, 2]. The biological roles attributed to EV are ever-increasing, placing EV research as a dominant field within immunology, hematology and cancer cell biology. Depending on their cell of origin, EV differ in protein and lipid composition, buoyant density, and biochemical and structural properties [1–3]. In addition, mechanisms of their formation and release from parental cells are crucial parameters for the classification of EV [3–5]. Together with expanding knowledge in the field of EV research, controversies have arisen concerning the diversity and the physiological relevance of circulating EV [2]. For example, the number and type of circulating EV, their half-lives in biological fluids, and the roles of vesicles in thrombosis and cancer progression are still hotly contested [6–10].

Exosomes, one of the smallest EV fractions, are released from a large spectrum of living cells and typically range from 50–200 nm in diameter [1–3]. The inward budding of cell membranes inside intracellular endosomes leads to the formation of multi-vesicular bodies (MVB) which can then fuse with the cell membrane to release exosomes [1, 2]. A larger fraction of EV (100–1000 nm diameter), directly shed from the surface of a healthy cell's membrane, are the microvesicles (MV) [1, 11]. Cells undergoing apoptosis are also known to release different types of EV that may range from 0.1–5 μm in diameter [12, 13]. The apoptotic fractions of EV that share a similar size range to MV, termed apoptotic vesicles (ApoV), display immunogenic activity [11, 14, 15]. Unlike exosomes, MV and ApoV are generated by the outward budding of the plasma membrane facilitated by the externalization of membrane phosphatidylserines (PS) [11, 16].

While EV release from primary cells may require activation, transformation, immortalization, or the initiation of cell death, most tumor cell lines studied constitutively release EV [4, 12, 17–28]. Tumor-derived EV are well known for their diverse biological functions, including immune suppression/activation, angiogenesis, tumor metastasis [20, 29–32], and are additionally implicated in triggering the coagulation cascade [10, 33, 34]. Moreover, tissue factor (TF) expression and PS exposure contributes to EV procoagulant activity [10, 23, 34, 35]. The four-fold increased risk of developing venous thromboembolism (VT) in cancer patients may be linked to the formation of tumor, or normal host tissue-derived EV [36, 37]. In particular, the fact that this risk is increased to around six-fold in patients receiving chemotherapy implicates tumor-derived ApoV [38]. However, the pathogenicity of chemotherapy-associated thrombosis remains poorly understood. Our study set out to clarify the phenotype of EV released from a well characterized tumor cell line, to investigate the potential for EV types to contribute to cancer-induced thrombosis and anti-cancer immunity. Of these vesicles, ApoV generated following the exposure of a tumor cell to chemotherapy are the least studied tumor-derived EV.

Using murine B16 melanoma as a model, we compared and characterized exosomes, MV, and ApoV, with respect to their size, morphology, molecular composition, and their ability to induce coagulation and immunity. We generated a distinguishable expression panel for the three EV fractions using various parameters such as tetraspanins, integrins, sialic acids, PS, heat shock proteins and histones. Importantly, we show that ApoV are released in greater quantities from dying cells compared to MV and exosomes from living cells. Membrane-derived EV (i.e. MV and ApoV) were superior at triggering thrombin and fibrin production via the extrinsic TF pathway of the coagulation cascade. Antigen-pulsing experiments showed that ApoV represent the most immunogenic fraction of EV and were able to provide long term protection of mice against B16 tumor challenge.

## RESULTS

### Purification and sizing of EV

**Figure 1:**
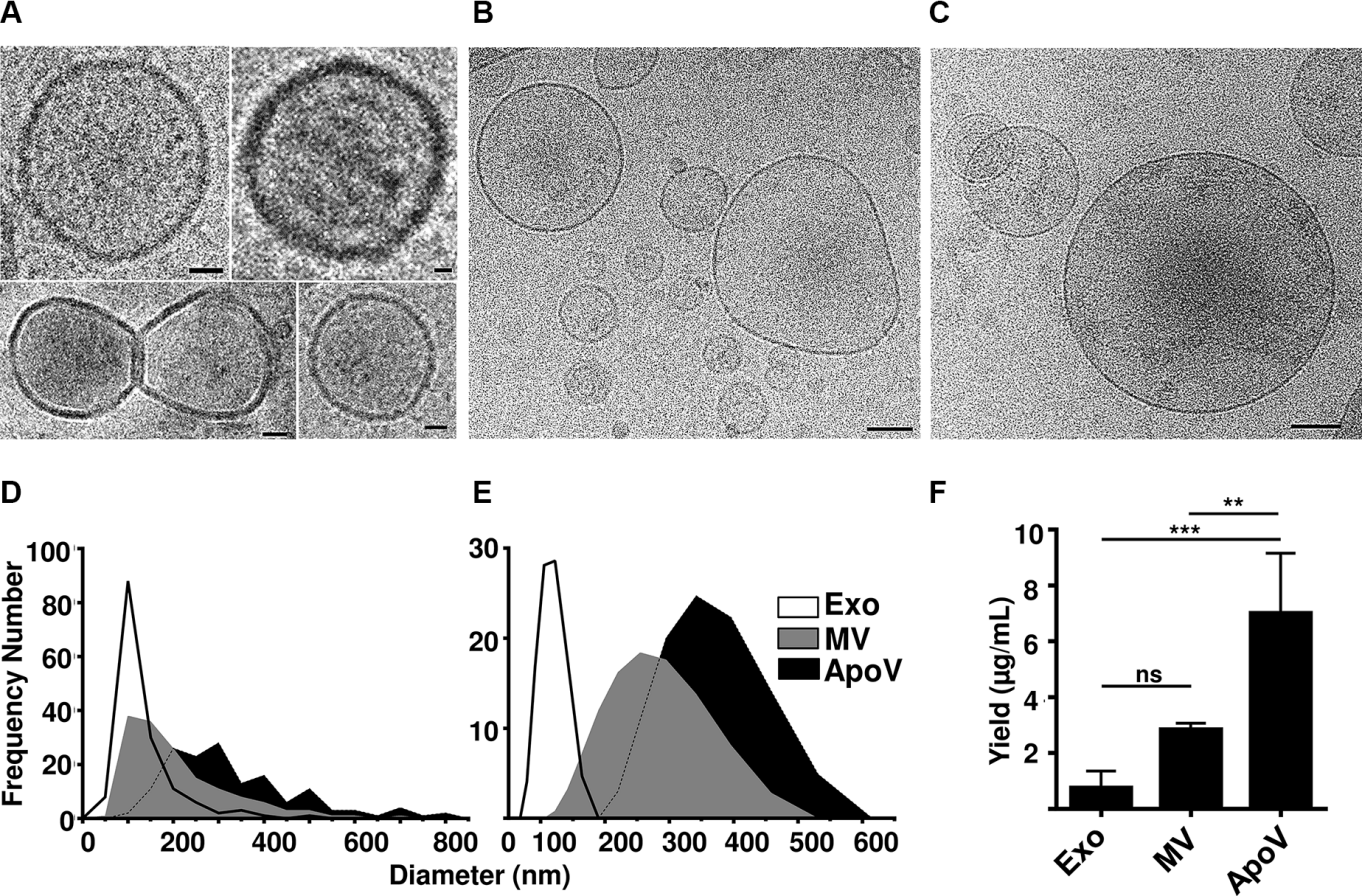
Cryo-electron microscopy, size range and yield of B16-F1-derived vesicles (**A**) Exosomes (Exo), (**B**) microvesicles (MV) or (**C**) apoptotic vesicles (ApoV) were purified by differential centrifugation of cell supernatant from live or doxorubicin-treated B16-F1 cells and extracellular vesicles (EV) visualized by cryo-electron microscopy. Original magnification ×20,000. Bars represent 20 nm (for Exo), and 100 nm (for MV and ApoV). Diameter of all three vesicle types determined by (**D**) cryo-electron microscopy (*n* = 150 vesicles) with a bin width of 5 nm and by (**E**) dynamic light scattering. (**F**) Yield of Exo, MV, and ApoV per mL of tissue culture; error bars represent mean ± SD. One-way ANOVA with Bonferroni post-correction test performed: ns = not significant; **P < 0.01, ***P < 0.001. Results are representative of three experiments.

We first visualized purified B16-F1-derived EV using cryo-electron microscopy (cryoEM; Figure 1A–1C; exosomes composite image). As shown in Figure 1D, exosomes were the smallest of vesicles exhibiting a diameter range of 51–478 nm (geometric mean; GM 135.5 nm ± 65.5 SD), MV had a range of 67–677 nm (GM 210 nm ± 114 SD), and ApoV had a range of 103–816 nm (GM 330 nm ± 147 SD). Similar values were determined using dynamic-light scattering (DLS): exosomes; range of 78–164 nm (GM 117.4 nm ± 32 nm); MV range 122.4–459 nm (GM 258.6 nm ± 112.8 SD), and ApoV 220–531 nm (GM 357 nm ± 112.1 SD; Figure 1E). The yield of ApoV, as determined by protein, was consistently higher than the other EV per mL of culture fluid (Figure 1F).

### Mass spectrometry and flow cytometry reveal a distinguishable panel for ApoV, MV, and exosomes

**Figure 2:**
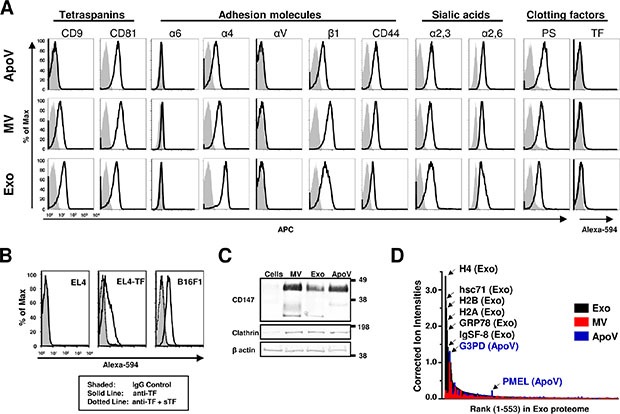
Surface expression comparison of B16-F1-derived vesicles using flow cytometry (**A**) Vesicles conjugated to aldehyde-sulfate microspheres were analyzed by flow cytometry using biotinylated (bio) antibodies for the indicated tetraspanins, adhesion molecules, and clotting factors; bio-annexin V for phosphatidylserine (PS) and goat anti-mouse tissue factor (TF). (**B**) TF expression on B16-F1 cells, EL4, or TF-transfected EL4 (EL4-TF) as analyzed by flow cytometry. Soluble TF (sTF) was used to neutralise the anti-TF polyclonal antibody. Biotin was detected using streptavidin-allophycocyanin (SA-APC), and TF was detected using rabbit anti-goat IgG Alexa Fluor^®^ 594. Grey shaded peaks represent BSA-bead control, goat IgG control for TF bead samples; black lines represent EV-beads or cells; dotted lines represent TF antibody neutralized cells. (**C**) Vesicle lysates were subjected to PAGE and Western blotted with goat anti-mouse CD147 (detected with anti-goat IgG-horse radish peroxidase (HRP), mouse anti-mouse clathrin heavy chain, and mouse anti-mouse β actin IgG-HRP (detected with anti-mouse IgG-HRP). MW in kDa are shown. Results are representative of at least two experiments. (**D**) TOP3 precursor ion intensities [69] normalised to β-actin (y-axis) are represented in rank order (x-axis) in the exosome proteome for the three vesicle types.

To further characterize B16-F1-derived EV, we analyzed their surface for a number of proteins including tetraspanins, adhesion molecules such as integrins and CD44, sialic acids, and the clotting factors TF and PS by flow cytometry (Figure 2A). Sucrose cushion-purified exosomes, MV and ApoV were dialyzed (10,000 Da cut off) and analyzed by mass spectrometry using a LTQ Orbitrap XL mass spectrometer (Table 1 and Supplementary Table 1). As illustrated in Figure 2D, EV differed in the abundance of a number of proteins, with particular enrichment of histones and heat shock proteins in exosomes, as compared to MV and ApoV. Notably, the ten most abundant ion scores in exosomes included the histones (H2A, H2B, H3.1 and H4), heat shock proteins (GRP78 and HSC71) and the tetraspanin CD81. Only ApoV showed enrichment for the melanoma tumour-associated antigen PMEL (Figure 2D and Supplementary Table 1). The raw data for the total 553 proteins identified by mass spectrometry are represented in Supplementary Table 1. By both flow cytometry and mass spectrometry, ApoV showed low expression of the tetraspanin protein CD9, while MV exhibited intermediate CD9 expression and exosomes the highest CD9 expression. All three EV were positive for the tetraspanin protein CD81 (Figure 2A), but with higher CD81 ion intensities obtained for exosomes, as compared to MV and ApoV (Table 1). In terms of integrin molecules, EV did not express detectable αV subunits. Exosomes showed the highest expression for the α4 subunit followed by MV, then ApoV. The β1 integrin subunit was highest in MV followed by exosomes, then ApoV (Figure 2A and Supplementary Table 1). While low levels of α6 were detected in all three vesicles, MV showed the highest expression for α6 (Figure 2A and Supplementary Table 1). Although positive, no differences in CD44 expression levels were detected between the three EV. All three EV showed high level sialic acid expression, which may contribute to the capture of extracellular vesicles in lymphoid tissue [39]. ApoV showed the highest level of PS expression followed by MV, then exosomes. Because the levels of TF were below the detectable limit for three EV, we confirmed the functionality of the TF-specific polyclonal antibody by labeling TF-transfected EL4, as well as the parental B16-F1 line, in the presence or absence of soluble TF-Fc protein. The results showed detectable TF expression on EL4-TF and parental B16-F1 cells and the specificity of the labeling was demonstrated by a loss in binding of the antibody to cells in the presence of excess, soluble TF-Fc protein (Figure 2B). The vesicular nature of our preparations was confirmed by the presence of CD147 and the coat protein clathrin by western blot and proteomic analysis. As expected from previous reports [25, 26, 40], there was a preferential association of CD147 and clathrin with vesicles, as compared to the B16 parental cell line (Figure 2C, Table 1 and Supplementary Table 1).

**Table 1:** Proteomic analyses of B16-derived extracellular vesicles (top 50 scores)

Accession (gi)	Protein Description	Exo	MV	ApoV
568893484	histone H4	3.37	0.14	0.08
31981690	heat shock cognate 71 kDa protein	2.70	0.22	0.09
28316760	histone H2B type 1-B	2.49	0.10	0.05
256773209	histone H2A.V	2.14	0.08	0.03
254540166	78 kDa glucose-regulated protein precursor	1.87	0.12	0.06
755495449	immunoglobulin superfamily member 8 isoform X1	1.42	0.14	0.06
148277591	syntenin-1 isoform 2	1.41	0.10	0.02
755502152	LOW QUALITY PROTEIN: uncharacterized protein LOC105244409	1.27	0.15	0.01
30061339	histone H3.2	1.25	0.04	0.00
19526794	CD81 antigen	1.20	0.11	0.06
755490905	LOW QUALITY PROTEIN: ubiquitin-40S ribosomal protein S27a-like isoform X1	1.01	0.20	0.08
6671509	actin, cytoplasmic 1	1.00	1.00	1.00
149273202	glyceraldehyde-3-phosphate dehydrogenase	1.00	0.42	1.30
568901980	MLV-related proviral Env polyprotein-like	0.99	0.22	0.06
755511141	MLV-related proviral Env polyprotein	0.98	0.21	0.06
755496111	LOW QUALITY PROTEIN: uncharacterized protein LOC105244006	0.79	0.44	0.06
16716569	protease, serine, 1 precursor	0.68	0.45	0.41
51491845	clathrin heavy chain 1	0.50	0.07	0.10
6755901	tubulin alpha-1A chain	0.46	0.12	0.26
6679439	peptidyl-prolyl cis-trans isomerase A	0.43	0.09	0.16
258547156	programmed cell death 6-interacting protein isoform 3	0.41	0.04	0.01
6680297	dnaJ homolog subfamily A member 1	0.40	0.01	0.00
45504394	integrin beta-1 precursor	0.39	0.12	0.08
7106439	tubulin beta-5 chain	0.38	0.09	0.17
568907654	tubulin alpha-4A chain isoform X1	0.37	0.12	0.20
126032329	elongation factor 1-alpha 1	0.35	0.20	0.43
171846253	transmembrane glycoprotein NMB precursor	0.34	0.02	0.06
21450277	sodium/potassium-transporting ATPase subunit alpha-1 precursor	0.33	0.32	0.26
568960833	pyruvate kinase PKM isoform X1	0.32	0.11	0.28
755509256	alpha-enolase isoform X1	0.31	0.10	0.18
22165384	tubulin beta-4B chain	0.30	0.05	0.12
13430890	histone H1.4	0.30	0.01	0.00
269914154	uncharacterized protein LOC239673	0.29	0.05	0.03
238637279	4F2 cell-surface antigen heavy chain isoform b	0.28	0.24	0.15
114326554	integrin alpha-4 precursor	0.28	0.06	0.04
226874906	14-3-3 protein epsilon	0.27	0.06	0.05
6677813	40S ribosomal protein S8	0.25	0.02	0.01
6753060	annexin A5	0.24	0.08	0.23
9845257	histone H1.2	0.24	0.02	0.00
755550334	14-3-3 protein zeta/delta isoform X1	0.23	0.05	0.05
9789937	dnaJ homolog subfamily A member 2	0.23	0.02	0.01
6680744	sodium/potassium-transporting ATPase subunit beta-3	0.23	0.18	0.16
116014342	basigin isoform 2 precursor	0.22	0.22	0.16
568917832	14-3-3 protein beta/alpha isoform X1	0.21	0.04	0.05
755506225	CDK5 regulatory subunit-associated protein 2 isoform X10	0.20	0.04	0.05
31543976	14-3-3 protein gamma	0.20	0.05	0.05
6756037	14-3-3 protein eta	0.19	0.04	0.05
17647499	hemoglobin subunit beta-2	0.19	0.04	0.07
60687506	fructose-bisphosphate aldolase C	0.19	0.09	0.10
568962761	guanine nucleotide-binding protein G(i) subunit alpha-2 isoform X1	0.19	0.11	0.06

Sucrose cushion purified B16F1-derived apoptotic vesicles (ApoV), microvesicles (MV), and exosomes (Exo) were subjected to mass spectrometry and data processed and searched against the mouse reference sequence database using the MASCOT, Sequest HT, and MS Amanda search engines. The TOP3 precursor ion intensities [69] normalised to β-actin of the highest 50 protein intensities present in the exosome samples are represented for all EV types. Additional proteomic data for the total 553 proteins identified in the three EV types are shown in Supplementary Table 1.

### Fibrin generation potential of EV

**Figure 3:**
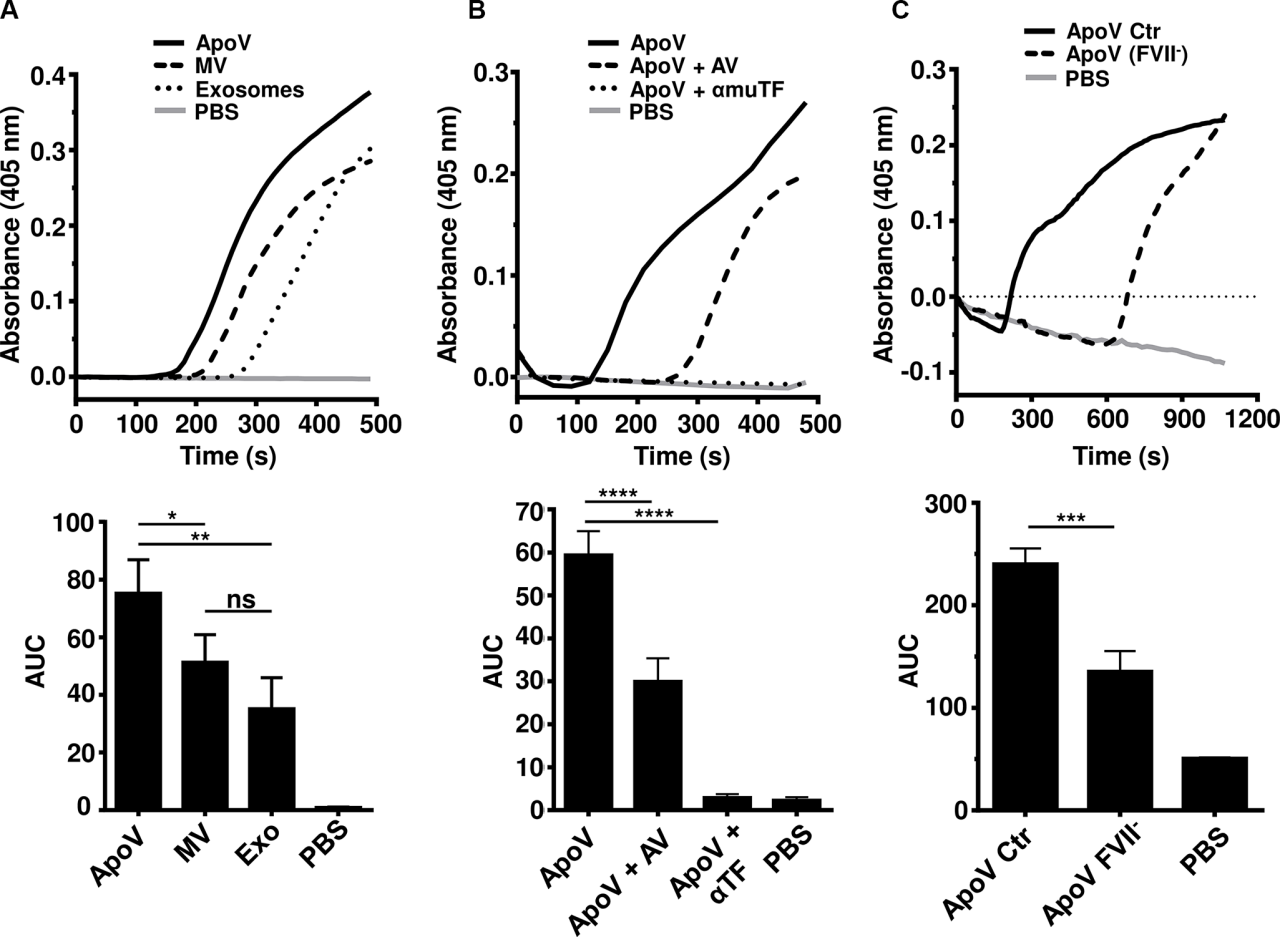
Fibrin generation assays comparing B16-F1-derived EV Equal amounts of purified EV (15 μg) were added to platelet-poor plasma (PPP) supplemented with CaCl_2_ to initiate coagulation. Fibrin generation was monitored at 405 nm until the negative control (PBS) started to generate detectable fibrin. (**A**) Fibrin generation induced by B16-F1-derived ApoV, MV, and Exo. (**B**) Fibrin generation assay of ApoV with inclusion of 20 μg/mL anti-mouse TF or 100 μg/mL annexin V (AV) to block PS. (**C**) Fibrin generation assay of ApoV using factor VII depleted plasma (FVII^−^) and commercial PPP (Ctr). Error bars represent mean ± SD. One-way ANOVA with Bonferroni post-correction test performed on the area under the curves (AUC): ns = not significant; *P < 0.05, **P < 0.01; ***P < 0.001, ****P < 0.0001. Samples were loaded in triplicate. Results are representative of at least three experiments.

Since tumor-derived EV have been implicated in cancer-related thrombosis, we next determined the procoagulant potential of the three types of EV using a fibrin generation assay (Figure 3A). ApoV generated significantly more fibrin as compared to MV and exosomes. Based on normalized protein content, exosomes were the least coagulative of the EV types. Despite the inability to detect TF by flow cytometry (Figure 2A), the activity of procoagulant ApoV was inhibited by anti-TF antibody and annexin V (Figure 3B). Furthermore, the importance of TF/extrinsic pathway, was confirmed using coagulation factor VII depleted (FVII^−^) plasma (Figure 3C).

### Density fractionation of ApoV

**Figure 4:**
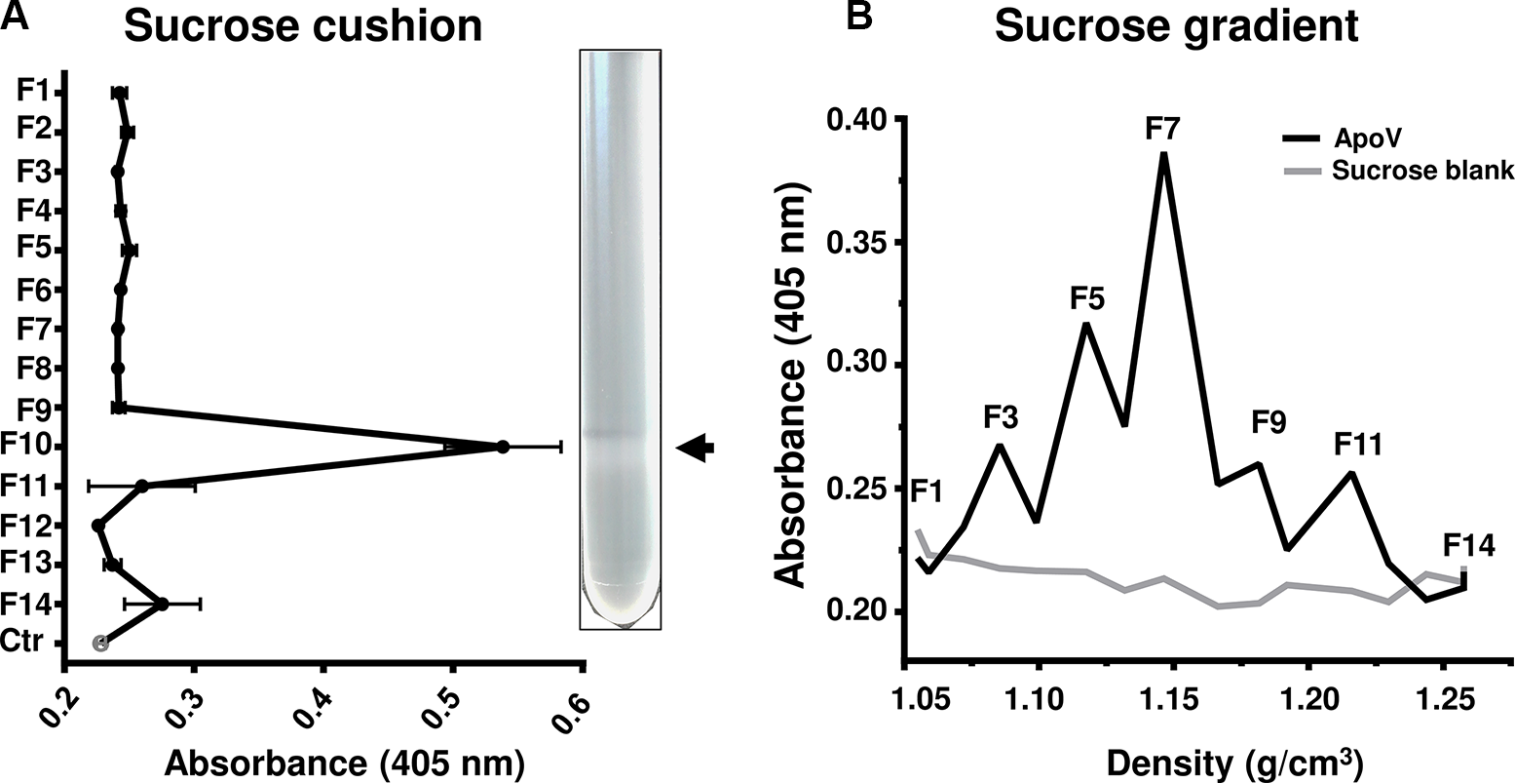
Fibrin generation assays of sucrose purified B16-F1-derived ApoV Following differential centrifugation, B16-F1 ApoV were further purified using sucrose gradients. The fractions (F1–14) were tested for in the fibrin generation assay, monitoring absorbance at 405 nm for 500 s. (**A**) ApoV purified in a 30% sucrose cushion/D_2_O. Arrow indicates the 0.21 g/cm^2^ interface of the sucrose cushion. Vesicle-free 30% sucrose/D_2_O was used as a control (Ctr); error bars represent mean ± SD. (**B**) ApoV purified by a linear sucrose gradient. For each experiment, 20 μL of each fraction was assayed. Results are representative of two experiments.

To rule out that contaminating macromolecules were contributing to the procoagulant activity of melanoma EV, we further purified the highly procoagulant ApoV fraction using a 30% sucrose/D_2_O cushion and assayed fractions by fibrin generation assay (Figure 4A). Only the ρ ≤ 0.21 g/ml interface exhibited procoagulant activity making it unlikely that non-vesicle associated proteins, polyphosphates or nucleic acids contributed to the observed fibrin generation initiated by ApoV. We next subjected ApoV to a continuous sucrose gradient and tested different density fractions for fibrin generation (Figure 4B). Although there was some heterogeneity within the coagulative ApoV fractions, the 1.12–1.15 g/cm^3^ density range encompassed the most procoagulant fractions.

### Thrombin generation potential of EV

**Figure 5:**
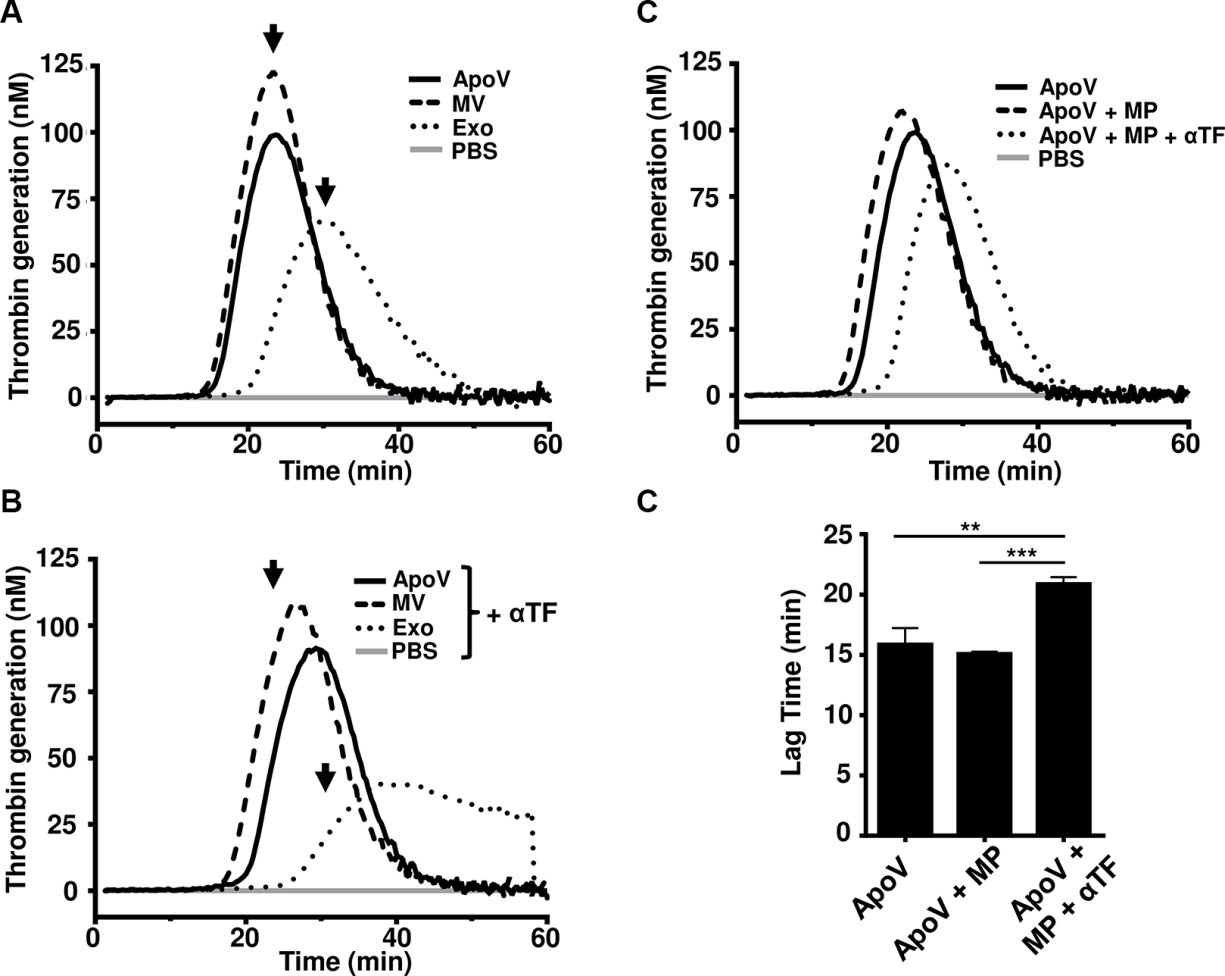
Thrombin generation assays B16-F1-derived EV Platelet-poor plasma was incubated with 2.5 μg of the indicated EV and the reaction was initiated by the addition of FluCa reagent. (**A**) Thrombin generation assay of the ApoV, MV, and Exo. (**B**) Thrombin generation by three EV types blocked with 20 μg/mL anti-mouse TF antibody (αTF). Arrows indicate peak thrombin values for MV, ApoV, and Exo obtained in absence of anti-TF antibody. (**C**) Thrombin generation by ApoV with the addition of lipid microparticles (MP) ± TF. (**D**) Bar graphs represent statistical analyses between samples in terms of lag time. One-way ANOVA using Bonferroni's multiple comparisons test was performed: **P < 0.01; ***P < 0.001. Samples were loaded in triplicates. Results are representative of two experiments.

To further confirm the procoagulant ability of the three B16-F1-derived EV types, we subjected purified vesicles to the thrombin generation assay (TGA; Figure 5A). Although results indicate that ApoV and MV were faster than exosomes at generating thrombin, significance between samples was not seen (Mean lag time: ApoV 15.89 min ± 1.34 SD; MV 15.89 min ± 0.16 SD; exosomes 20.22 ± 4.96 SD). Similar to our fibrin generation assay (Figure 3), thrombin generation was retarded for all EV by the inclusion of neutralizing anti-TF antibody in the TGA (Figure 5B). To ensure that phospholipid was not limiting for the activity of TF, we supplemented the reaction with phospholipid microparticles (MP; Figure 5C and 5D). Although only a small decrease in lag time to peak thrombin production was noted following the addition of phospholipid, blocking TF in the presence of excess MP significantly reduced thrombin generation capability of ApoV.

### Anti-cancer responses induced by EV

**Figure 6:**
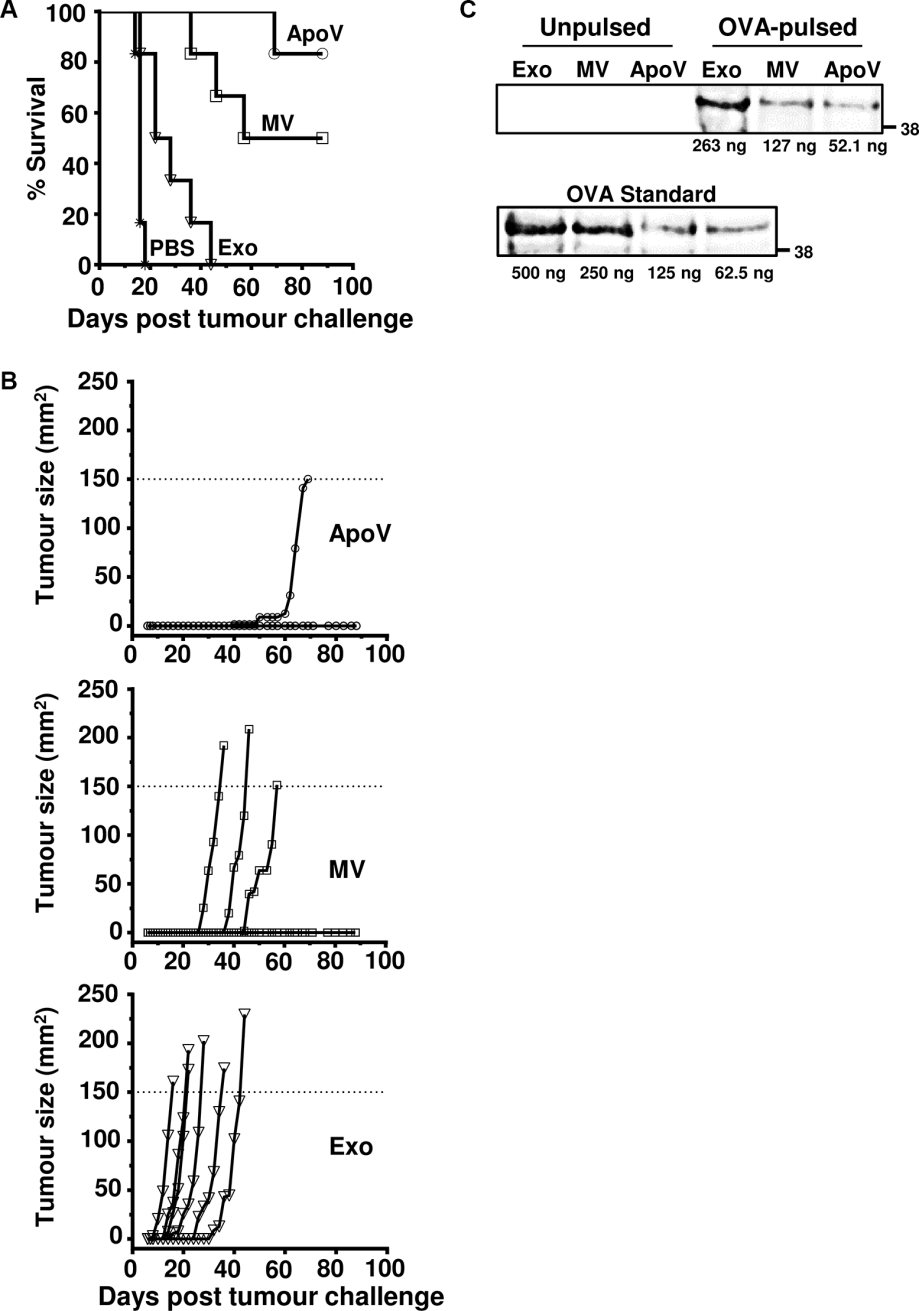
The anti-tumor effects of exogenously-pulsed B16-OVA vesicles on tumor growth Mice were injected subcutaneously with ApoV, MV, Exo (isolated by differential centrifugation of ovalbumin-pulsed B16-OVA-derived vesicles), or PBS in the flank before challenged with B16-OVA cells at the opposite flank seven days later. Tumor size was recorded until tumors reached 150 mm^2^. (**A**) Percent survival of the four mouse groups (*n* = 6 mice/group). (**B**) Tumor size represented for individual mice. ApoV/Exo (*P* < 0.0005); ApoV/MV (not significant); MV/Exo (*P* < 0.0022); PBS/Exo (*P* < 0.004) by Mantel-Cox test. The protective results for ApoV were confirmed in an additional experiment. (**C**) OVA-pulsed Exo, MV or ApoV were probed by western blotting with rabbit anti-OVA and detected with donkey anti-rabbit IgG-DyLight-800. A titrated OVA standard was used to quantify the amount of OVA in each sample. Note, endogenously expressed ovalbumin in the B16-OVA cell line (‘Unpulsed’) is below the limit of detection by western blotting. One of two experiments performed.

To determine if the three EV types could play a role in inducing immunity against the B16 tumor we first immunized mice subcutaneously (s.c.) with EV derived from B16-ovalbumin (B16-OVA) in the flank. We then challenged all mice, seven days later, with B16-OVA cells at the opposite flank. Although the B16-OVA cell line expresses ovalbumin at sufficient levels to act as a surrogate tumor antigen for protection in ovalbumin-vaccinated mice [41, 42], no protection was observed when exogenously supplied ovalbumin was omitted from the vaccine B16-OVA ApoV preparations (data not shown). B16-OVA cells were therefore treated with additional soluble ovalbumin (200 μg/ml) prior to the isolation of vesicles. Mice immunized with OVA-pulsed ApoV showed the highest protection with only one mouse developing a B16-OVA tumor that reached maximum size at day 69 (Figure 6A–6B). This level of protection was followed by the mice immunized with OVA-pulsed MV, where three mice reaching maximum tumor size at days 36, 46, and 57. All mice immunized with OVA-pulsed exosomes reached maximum B16-OVA tumor sizes at 16, 22, 28, 36, and 44 (Mantel-Cox analysis; MV vs. exosomes: *P* < 0.0022; ApoV vs. exosomes: *P* < 0.0005; ApoV vs. MV; no significance). Surprisingly, the weakly protective exosomes contained a greater quantity of ovalbumin, as compared to MV and ApoV (Figure 6C). Therefore, the superior protection afforded by ApoV was not a result of enhanced ovalbumin loading into this EV subtype.

## DISCUSSION

EV released from living and dying tumor cells contribute to the outcome of cancer progression in the host. For example, EV have been proposed to induce a pre-metastatic niche for cancer metastasis [4, 20, 22, 43] and contribute to thrombotic events, such as pulmonary embolism deep vein thrombosis in cancer patients [10, 21, 33, 34, 36–38, 44]. EV-associated TF is responsible for the prothrombotic effect of EVs in a mouse model of thrombosis [34]. Less well studied is the role of tumor-derived EV in the induction of immune responses to the tumor itself.

Our results offer clarity of the distinction between vesicles released from the endosome and plasma membrane both from living cells and under chemotherapeutic stress. The murine B16 cell line was chosen for this study as it is the most frequently used syngeneic tumor mouse model studied and recapitulates features of human melanoma including immune suppression and metastasis, and a variable response to immunotherapy [45].

Our study identified distinct morphological and phenotypic features of EV. Exosomes, the smallest vesicle type analyzed, displayed a similar size range to those previously reported for the B16 cell line [46]. ApoV displayed the largest size and greatest range in diameter, as compared to MV and exosomes (Figure 1). B16 melanoma-derived ApoV were significantly larger than ApoV released from the EL4 lymphoma cell line, as determined in our recent study [14], demonstrating that parental cell type may predetermine certain physical attributes of EV. DLS generates a monomodal distribution and the software assumes that particles are spherical and non-aggregated, while the cryoEM sizing technique employed was simple diameter measurement of individual vesicles at the widest point. While a small difference in vesicle sizing was noted between the DLS and cryoEM techniques, the geometric means were in fact quite similar. The reason for the size difference between the two plasma membrane-derived EV (MV and ApoV) is not entirely clear, but likely relates to caspase activation during the apoptotic response to doxorubicin. The mechanism of release of MV and ApoV are thought to be fundamentally similar, starting with asymmetric redistribution of membrane phospholipids, including the translocation of phosphatidylserine to the outer leaflet, followed by the budding process via actin-myosin interactions [11]. However, in apoptotic vesicle formation, actin-myosin interactions are dependent on caspase cleavage of Rho-associated kinases 1 (ROCK1) which in turn phosphorylates the myosin light chain for bleb expansion [47].

Phenotypically, major distinctions in terms of molecular profiles were noted between membrane-derived (MV and ApoV) vesicles as compared to exosomes. Melanoma exosomes were strikingly enriched in histones and heat shock proteins, as compared to the other two EV types. Remarkably, this finding is similar to an earlier comparison of apoptotic vesicles and exosomes released from mouse dendritic cells, and is also consistent with the exosome proteome of human dendritic cells [5, 12] or murine and human melanoma-derived exosomes [43, 48]. Historically, the histone content of EV was thought to be via contamination with apoptotic bodies [12]. However, histones are present in both cytoplasmic and nuclear pools [49] and frequently appear in exosome preparations [5, 12, 43, 48]. Furthermore, we consider that contamination of apoptotic vesicles into our exosome preparations was quite unlikely for the following reasons: (i) our exosome preparations were pre-depleted of cells, debris and MV using sequential 450 × g, 3200 × g and 25,000 × g steps then filtered through a 0.2 μm filter and floated over a sucrose/D_2_O cushion prior to dialysis, and (ii) histones were present in the MV or ApoV fraction at ion intensities at least 20–65 fold lower than exosomes (Supplementary Table 1). It is surprising, given the highly positive charge of histones, that more links have not been investigated between the RNA and histone cargo of exosomes. We suggest that the enrichment of histones in exosomes may reflect a chaperone role histones for the nucleic acid content of exosomes [1, 2] and it is likely that RNA-histone interactions will become an active area of future research. Indeed, a direct association of miRNA and histones has been reported for breast cancer cell line-derived exosomes [50] and histone H3 modification was suggested to be essential for exosome release [51].

In cancer patients, tumor vesicles have been implicated in thrombosis, metastatic spread and immune suppression [10, 20–22, 25, 29, 33, 37, 38, 43, 44]. Our investigation into the relative procoagulant activity of vesicles revealed a superior ability of ApoV to induce fibrin generation in platelet poor plasma, compared to MV and exosomes. However, in the TGA, the difference between ApoV and MV was less marked and failed to reach significance. Nevertheless, in both the fibrin generation and TGA, the plasma membrane-derived ApoV and MV displayed higher levels of procoagulant activity compared to exosomes. Although the fibrin generation and TGA differ dramatically in duration (approximately 8 min vs. 25 min to peak fibrin or thrombin respectively) the same pattern was observed; MV and ApoV were more procoagulant than exosomes (Figures 3 and 5). It should be noted that that fibrin clot time precedes the peak thrombin production, with < 1% of total thrombin production required for clot formation [49, 52]. Interestingly, although extracellular histones have been shown to enhance thrombin generation in platelet-poor plasma, exosomes, which contained the highest content of histones, were the least procoagulant of the three EV types [53].

Despite the fact that EV TF was below the detectable limit on EV analyzed by flow cytometry (Figure 2), we were nevertheless able to show its importance by neutralization of TF function, or by removing its critical ligand FVII / FVIIa in our fibrin generation assay (Figure 3B and 3C). Fibrin generation was not completely inhibited in FVII-depleted plasma. However, the commercial source does not guarantee complete removal of FVII by affinity chromatography. Thus residual clotting in commercial FVII plasma may reflect residual FVII activity, rather than the initiation of alternate pathways of coagulation by ApoV. Although TF was not detectable by flow cytometry, it was still highly functional. It should be noted that only picomolar concentrations of TF are required for high level activity in the TGA [54]. Therefore, the activity of TF resembles that of cytokines, which are able to induce large biological activity at low molar concentrations. For example, only 3–10 TF molecules per μm^2^ are sufficient to induce fibrin deposition under flow conditions [55, 56]. Interestingly, ApoV procoagulant activity far exceeds that of parental tumor cell lines, including those overexpressing TF, when normalized for protein content (manuscript in preparation). Therefore, the activity of TF is likely critically dependent on the context of anionic phospholipids, particularly phosphatidylserine [57]. which was enriched on ApoV relative to MV and exosomes (Figure 2). More controversially, TF activity may require decryption through dimerization or disulfide bond formation [58]. A caveat with our conclusions on the relative quantity and activity of TF on vesicles is the semi-quantitative nature of the highly sensitive flow cytometric assay employed. For example, the same ligand density on the membranes of larger flow cytometric events (e.g. tumor cells) would generate higher fluorescent signals, as compared to smaller particles (i.e. 4 μm beads with immobilized EV). In addition, autofluorescence and non-specific binding of isotype controls may mask detection on antibody ligands on the EV preparations.

Due to genomic and epigenetic alterations, tumor can express neoantigens, or overexpress antigens, that are recognized by the immune system [30]. Additionally, tumor associated antigens and endogenous adjuvants are translocated to vesicles [15, 28, 59]. Notably, the release of tumor antigen on EV is one of the major pathways of the induction of immune responses against tumors [28]. In our experiments, challenge of mice with B16-OVA demonstrated that ApoV afford the highest anti-tumor protection, as compared to MV and exosomes. The exceptional ability of ApoV to protect against melanoma challenge could relate to products of “immunogenic cell death”, such as high mobility group box protein B1 (HMGB1) and calreticulin translocating to the ApoV pathway [59, 60]. However, neither HMGB1 or calreticulin were present in the ApoV proteome (Supplementary Table 1), suggesting that other as yet unidentified factors mediate the immunogenicity of ApoV. We cannot rule out a partial contribution of the PMEL tumour-associated antigen enriched in the ApoV proteome (Figure 2D) to the observed immune response, however ApoV failed to induce measurable anti-tumour effects in the absence of exogenously supplied ovalbumin. In addition, the products of apoptotic cell death are efficiently phagocytosed and processed by antigen presenting cells [61], with the likelihood that the abundant release of ApoV with high level expression of phosphatidylserine contributes to enhanced uptake and processing [24, 47, 60, 62]. Although less protective than ApoV, MV were significantly more protective than exosomes. It is possible that distinct factors mediate the protection observed with MV and ApoV, in which case a combination of MV and ApoV might induce even higher levels of protection compared to either vesicle fraction alone. However, it seems equally likely that ApoV and MV harbour the same immunogenic factors, but in different quantities, given the lower levels of protection induced by MV immunization, as compared to ApoV immunization. The poor level of protection afforded by exosomes is surprising given their well characterised ability to capture exogenously supplied antigens (see Figure 6C) and stimulate the immune response [1–3, 39, 63]. Nevertheless, our results with melanoma-derived exosomes closely match the level of protection afforded using ovalbumin-pulsed dendritic cell-derived exosomes with B16-OVA challenge at day 7 [64]. This may reflect a general lack of efficacy for exosomes within this particular melanoma experimental setting.

A weakness in our study was the inability to exclude the possibility that ApoV preparations might contain MV and exosomes released during the induction of cell death by doxorubicin. Conversely, MV preparations might be contaminated with ApoV resulting from normal cell turnover and death during cell culture. Such contamination might mask antigen phenotypic and functional differences between these EV preparations. Nevertheless, the techniques employed allowed for sufficient enrichment of the different EV preparations for the discernment of distinct yields, morphological, molecular, procoagulant and immunogenic properties between the three EV populations.

Our study is the first to directly compare the procoagulant and immunogenic properties of exosomes, MV and ApoV. In particular, this study highlights the contribution of abundantly produced ApoV to pathological states, and their contribution to the anti-cancer response. The greater yield of ApoV released from the cell, as compared to exosomes and MV, further emphasizes the potential of ApoV to contribute to the pro-thrombotic state of cancer patients. Cytoablative anti-cancer therapy may therefore enhance the risk of thrombotic events, but also enhance T cell and natural killer cell-mediated anti-cancer responses via immunogenic EV release.

## MATERIALS AND METHODS

### Cell culture

The C57BL/6 derived melanoma cell lines B16-F1 (ATCC, Manassas, VA) and B16-OVA (B16-F0 cell line transfected with the full-length ovalbumin (OVA) gene from Edith Lord, University of Rochester, NY [65]), and C57BL/6 derived thymoma derived EL4 cell line (ATCC, Manassas, VA) were cultured at 37°C with 5% CO2 in R5 (RPMI-1640 (Gibco #31800-022) supplemented with 5% fetal bovine serum (FCS; PAA Laboratories, Austria), 55 μM β-mercaptoethanol (Gibco #21985-023), 100 U/mL penicillin (Gibco #15140-122), 100 μg/mL streptomycin (Gibco #15140-122), and 2 μg/mL NaHCO3. Stable murine TF transfectants were produced by transfecting EL4 cells with a pcDNA3.1+ vector containing an Apa I insert of full length murine TF gene (encoding amino acids 1-294; NM_010171; synthesized by Genscript; Piscataway, NJ), followed by selection with G418. For exosome preparation, 50% FCS in RPMI was spun at 100,000×g overnight at 4°C to deplete endogenous EV.

### Vesicle preparation

B16-F1 or B16-OVA cells were incubated for 48 hours either with (ApoV) 25 μM doxorubicin (Baxter Healthcare Ltd, NZ) or without (MV and exosomes) doxorubicin at 70% confluence (approximately 1 × 10^5^ cells/mL) in exosome-depleted R5 at 37°C with 5% CO_2_. In order to obtain vesicle-rich fractions, the supernatant was first depleted of cells and debris using differential centrifugation at 450 × g for five minutes, followed by 3200 × g for 20 minutes at 4°C in a 225 mL conical tube (Falcon, *In Vitro* Technologies, Auckland, NZ). MV (from untreated cultures) or ApoV (doxorubicin treated cultures) enriched fractions were then pelleted by centrifugation at 25,000 × g for one hour at 4°C. For exosomes, the supernatant was first depleted of larger vesicles at 25,000 × g for 1 hour. The remaining supernatant was then filtered using a 0.22 μm nitrocellulose filter (Cole-Parmer^®^ #EW-02915-52) and exosomes pelleted at 100,000 × g for 60 minutes at 4^°^C. All vesicles were washed twice in phosphate buffered saline (PBS; Gibco #21600-010). Where stated, vesicles were further purified by either a discontinuous sucrose cushion or a linear sucrose gradient. Briefly, for the sucrose cushion, vesicles were resuspended in 10 mL of PBS and overlaid onto 4 mL of 30% sucrose, 200 mM Tris/deuterium oxide; D_2_O, centrifuged at 100,000 × g for 75 minutes at 4°C and vesicles at the interphase aspirated (approximately 2 mL). Alternatively, for continuous gradient separation, the ApoV pellet was resuspended in 1 mL of 2.5 M sucrose/20 mM HEPES, pH 7.2, and then a linear sucrose gradient (0.25–2 M sucrose/20 mM HEPES, pH 7.2) was overlaid onto the ApoV suspension and centrifuged at 100,000 × g for 18 hours at 4°C. Fractions (1 mL) were removed, and their density determined with an Abbe refractometer (Tokyo, Japan). The protein content of purified vesicles was quantified using a Bradford assay [14].

### Sizing and cryo-electron microscopy analysis of vesicles

Purified vesicles (4 μL) were loaded onto plasma-glowed Quantifoil 2/2 grid and blotted to remove excess liquid. The grid was frozen by plunging into −180°C liquid ethane within a Reichert KF80 plunge freezing device (C. Reichert, Austria) and then stored in liquid nitrogen. The grids were then mounted into a pre-chilled Gatan 914 cryo holder and viewed in a JEOL 2200FS cryo-transmission electron microscope with an omega filter. Zero-loss images were acquired using a filter width of 20–25 eV via a TVIPS F416 camera. The diameters of the vesicles were measured using the software IMOD. Alternatively, vesicle size was measured using DLS (Zetasizer Nano-ZS; Malvern Instruments, UK).

### Flow cytometric analyses of vesicles

Ten micrograms (protein content) of purified vesicles (MV, exosomes, or ApoV) in 100 μL of PBS was added to 25 μL of aldehyde-sulfate beads (4 μm diameter; 1.4 × 10^9^ beads/mL; Invitrogen #A37304) and incubated rotating overnight at 4°C. The vesicle-conjugated beads were then blocked with 0.5 mL of 0.05% bovine serum albumin (BSA; Gibco #30063-572)/PBS for 15 minutes on ice, pelleted at 7000 × g at 4°C, then quenched with 0.5 mL of 100 mM glycine/PBS for 30 minutes on ice. Pelleted beads were then washed in 1 mL of 0.05% BSA/PBS and finally resuspended in 100 μL of 0.05% BSA/PBS. Vesicle-conjugated beads (3 μL/reaction) were then analyzed for α2,3- or α2,6-linked sialic acid expression with biotinylated (bio) lectins 5 μg/mL MAL-II (Vector #B-1265) or 0.5 μg/mL SNA-I (Vector #B-1305) respectively, or labeled with 5 μg/mL bio-rat anti-mouse CD9 (BD Pharmingen #558749), bio-hamster anti-mouse CD81 (BD Pharmingen #559518), allophycocyanin (APC)-conjugated rat anti-mouse CD49f (BioLegend #313615), bio-rat anti-mouse CD49d (BioLegend #103703), bio-rat anti-mouse CD51 (BioLegend #104103), bio-hamster anti-mouse CD29 (BD Pharmingen #555004), goat anti-mouse TF (R&D #AF3178) diluted in 0.05% BSA/PBS for 15 minutes on ice. Following washing with 0.05% BSA/PBS, biotin was detected using 1 μg/mL APC-conjugated streptavidin (BioLegend #405207), primary anti-TF antibody was detected with 2 μg/mL rabbit anti-goat IgG Alexa Fluor^®^ 594 (ThermoFisher #A-11080). Vesicle-conjugated beads were resuspended in 0.05% BSA/PBS and analyzed by flow cytometry (BD LSRFortessa; FlowJo). For bio-annexin V binding (BD Pharmingen #556418), annexin V binding buffer was used instead of 0.05% BSA/PBS. A murine TF-Fc (human IgG1) fusion protein was produced by fusing the human Ig kappa signal sequence to DNA encoding amino acids 29-251 from the murine TF extracellular domain. This insert was cloned in frame into the BamHI / NheI sites of pCMV-SPORT-Fc. TF-Fc was produced by transient transfection in HEK293 and purified by Protein A affinity chromatography. Where stated, 10 μg/mL soluble murine TF-Fc was pre-incubated with the primary anti-mouse TF antibody for 30 minutes on ice, prior to cell labelling.

### Western blotting

B16-F1 cells or EV fractions were lysed in lysis buffer (0.02% azide, 150 mM NaCl, 0.25% CHAPS, 0.5% Triton-X100, 100 mM Tris, pH 8.0 plus complete^™^ protease inhibitor; Roche #11-697-498-001). Lysates (10 μg in 10 μL) were added to 9 μL of NuPAGE LDS sample buffer (Invitrogen #NP0007) and 1 μL of NuPAGE sample reducing agent (Invitrogen #NP0009). Samples were boiled and reduced. Samples were then run on a NuPAGE 12% Bis-Tris gel (Invitrogen #NP0342BOX) in NuPAGE MOPS-SDS running buffer (NuPAGE #NP0001) plus 0.5 mL of antioxidant (Invitrogen #NP0005) for two hours at 150 V and 126 mA on ice. Electrophoresed samples were transferred to Amersham Protran nitrocellulose membrane (GE Healthcare #GE10600018) using 1 × NuPAGE transfer buffer (Invitrogen #NP0006) for one hour at 30 V and 170 mA on ice. Membranes were then blocked with 1% BSA/PBS for 1 hour, then incubated with either 0.5 μg/mL mouse anti-mouse β actin (Sigma #A1978), 1 μg/mL mouse anti-mouse clathrin heavy chain (BD Biosciences #610500), or 1 μg/mL goat anti-mouse CD147 (Santa Cruz #sc-9757) for 2 hours and washed thrice with 0.02% Tween20/PBS for 5 minutes each washing step. Primary antibodies were then detected with goat anti-mouse IgG-horse radish peroxidase (HRP; Sigma-Aldrich #A4416) for β actin and clathrin and rabbit anti-goat IgG-HRP (Sigma-Aldrich #A5420) for CD147. Secondary antibodies were diluted 1/1000 in 1% BSA/PBS and applied for 1 hour. Membranes were washed as described above, rinsed with milliQ H_2_O and HRP signal developed with diaminobenzidine (DAB) and H_2_O_2_ (Sigma-Aldrich #D-4293) in milliQ H_2_O. Reactions were stopped by rinsing with milliQ H_2_O. All incubations were performed at room temperature while rocking. For OVA protein quantification on B16-OVA-derived ApoV, MV, or exosomes, membranes were blocked with 0.1% caseinate/PBS for 2 hours, then incubated with 5 μg/mL rabbit polyclonal anti-OVA (Polysciences, Warrington, PA, USA #23744) for 2 hours. The primary antibody was then detected using donkey anti-rabbit IgG-DyLight-800 (Pierce #SA5-10044) diluted 1/10000 in 0.1% caseinate/PBS for 1 hour. Membranes were visualized with an Odyssey Fc imaging system (LI-COR, Lincoln, NE, USA). Quantification of OVA was performed using the Image studio Lite software (Lincoln, NE, USA) using titrated OVA (500, 250, 125, 62.5 ng) as a standard (Sigma-Aldrich #A5503).

### Proteomic analyses

EV prepared by differential centrifugation were further purified by 30% sucrose/D_2_O and dialyzed (10,000 Da cut off) against PBS to remove sucrose and D_2_O. Vesicle lysates were buffer exchanged and purified by the FASP (filter-aided sample preparation) method [66] using 0.5 ml ultrafiltration units with a molecular weight cut-off of 3 kDa (Millipore). Reduction, alkylation and tryptic protein digestion were performed on filter. The recovered tryptic peptides of each sample were then subjected to liquid chromatography coupled tandem mass spectrometry using an Ultimate 3000 uHPLC-system inline coupled to the nanospray source of a LTQ Orbitrap XL mass spectrometer. Raw data were processed through the Proteome Discoverer software (Thermo Scientific) and searched against the mouse reference sequence database (download Nov 2015 from http://www.ncbi.nlm.nih.gov/refseq; 57928 sequence entries) using the MASCOT (matrixscience.com), Sequest HT (Thermo Scientific) and MS Amanda [67] search engines. The Percolator algorithm [68] was used to adjust the score threshold to an estimated peptide false discovery rate of 1%. Only proteins with two significant peptide hits were considered as significantly identified. Relative protein abundances between the different samples were estimated through using the TOP3 approach [69]. The TOP3 intensity values were calculated using the Proteome Discoverer software and normalized to the β-actin ion intensity of each sample.

### Fibrin generation assay

Purified vesicles (quantities as stated) in 20 μL of PBS were added to 100 μL of citrated platelet-poor plasma (Siemens #10446238, or obtained from normal donors with the approval of the regional Human Ethics committee) in a 96-well plate. Coagulation was initiated by adding 10 mM CaCl_2_ and the absorbance at OD_405nm_ was measured every 30 seconds over 30 minutes at 23°C using a plate reader (Varioskan Flash, Thermo Scientific). All samples were performed in triplicates. Where stated, 20 μg/mL goat anti-mouse TF polyclonal antibody (R&D #8686) or 100 μg/mL annexin V (eBioscience #BMS306) were added for blocking experiments.

### Thrombin generation assay

Purified vesicles (quantities as stated) in 20 μL of PBS or 20 μL of thrombin calibrator (715 nM specific activity; Thrombinoscope #TS20.00) was added to 80 μL of citrated platelet-poor plasma (Siemens #10446238) in a 96-well plate. Henceforth, instructions were followed as per the Thrombinoscope software. Briefly, the plate was pre-warmed at 37°C in the Fluoroskan Ascent fluorometer (Thermo Scientific), 20 μL of FluCa (Fluo-Substrate containing calcium; 1:40; Thrombinoscope #TS50.00) dispensed and thrombin activity measured for 1 hour. Where stated, 20 μg/mL rat anti-mouse TF monoclonal antibody (1H1; Genentech, San Francisco, CA) was added for blocking experiments.

### Vesicle immunization and tumor implantation

B16-OVA was cultured in R5, pulsed for 48 hours with 200 μg/mL OVA protein (Sigma-Aldrich #A5503) and vesicles harvested as stated above. For generation of ApoV, doxorubicin was added simultaneously with the OVA protein. C57BL/6 mice were obtained from the Jackson Laboratory (Bar Harbor, ME) and bred and housed under specific pathogen-free conditions at the University of Otago Hercus-Taieri Research Unit as described [70]. All experiments were approved by the University of Otago regional animal ethics committee. Mice were immunized s.c. with 25 μg (in 50 μL of PBS) of B16-OVA-derived ApoV, MV, or exosomes in the flank. Seven days later, B16-OVA cells were harvested from logarithmically growing cultures using a cell scraper, filtered through a 70 μm cell strainer, and resuspended in PBS. Mice were then challenged with 1 × 10^5^ B16-OVA cells s.c. in the opposite flank to the immunization site. Tumor growth was determined by measuring the length and width using calipers every 1–2 days. Results are expressed as the mean product of the tumor diameters. Mice were removed from the study when tumors reached 150 mm^2^. Data are represented as tumor growth curves or as Kaplan-Meier survival plots using Graphpad Prism 6 (GraphPad, San Diego, CA).

### Statistical analysis

All statistical analyses were performed with GraphPad Prism 6 (GraphPad, San Diego, CA). Fibrin generation assays were analyzed using one-way ANOVA with Bonferroni post-correction test. Survival data were represented as tumor growth curves or as Kaplan-Meier survival plots and analyzed using the Mantel-Cox test.

## SUPPLEMENTARY MATERIALS TABLE




