# Supplementary material for: Procoagulant and immunogenic properties of melanoma exosomes, microvesicles and apoptotic vesicles
Source: Oncotarget. 2016 Jul 22;7(35):56279–94. doi: 10.18632/oncotarget.10783 (PMC5302914; doi:10.18632/oncotarget.10783)
Supplement: Supplementary file 1 [file oncotarget-07-56279-s001.pdf]

Procoagulant and immunogenic properties of melanoma exosomes, microvesicles and apoptotic vesicles

Supplementary Materials

Supplementary Table S1: Proteomic analyses of B16-derived extracellular vesicles

Sucrose cushion purified B16F1-derived apoptotic vesicles (ApoV), microvesicles (MV), and exosomes (Exo) were subjected to mass spectrometry and data processed and searched against the mouse reference sequence database using the MASCOT, Sequest HT, and MS Amanda search engines. The TOP3 precursor ion intensities [69] of the highest 553 protein intensities identified using the Proteome Discoverer software were normalized to β-actin ion intensities present in each sample. See Supplementary_Table_S1
